# Manual Dexterity and Aging: A Pilot Study Disentangling Sensorimotor From Cognitive Decline

**DOI:** 10.3389/fneur.2018.00910

**Published:** 2018-10-29

**Authors:** Loic Carment, Abir Abdellatif, Carmelo Lafuente-Lafuente, Sylvie Pariel, Marc A. Maier, Joël Belmin, Påvel G. Lindberg

**Affiliations:** ^1^Inserm U894, Université Paris Descartes, Paris, France; ^2^Plateforme de Recherche Clinique en Gériatrie, Hôpitaux universitaires Pitié-Salpêtrière-Charles Foix, APHP, Ivry-sur-Seine, France; ^3^Service de Gériatrie à orientation Cardiologique et Neurologique, Sorbonne Université, Hôpitaux Universitaires Pitié-Salpêtrière-Charles Foix, APHP, Ivry-sur-Seine, France; ^4^Département de soins ambulatoires, Hôpitaux universitaires Pitié-Salpêtrière-Charles Foix, APHP, Ivry-sur-Seine, France; ^5^FR3636 CNRS, Université Paris Descartes, Paris, France; ^6^Department of Life Sciences, Université Paris Diderot, Paris, France

**Keywords:** manual dexterity, sensorimotor integration, aging, cognitive decline, Alzheimer disease

## Abstract

Manual dexterity measures can be useful for early detection of age-related functional decline and for prediction of cognitive decline. However, what aspects of sensorimotor function to assess remains unclear. Manual dexterity markers should be able to separate impairments related to cognitive decline from those related to healthy aging. In this pilot study, we aimed to compare manual dexterity components in patients diagnosed with cognitive decline (mean age: 84 years, *N* = 11) and in age comparable cognitively intact elderly subjects (mean age: 78 years, *N* = 11). In order to separate impairments due to healthy aging from deficits due to cognitive decline we also included two groups of healthy young adults (mean age: 26 years, *N* = 10) and middle-aged adults (mean age: 41 years, *N* = 8). A comprehensive quantitative evaluation of manual dexterity was performed using three tasks: (i) visuomotor force tracking, (ii) isochronous single finger tapping with auditory cues, and (iii) visuomotor multi-finger tapping. Results showed a highly significant increase in force tracking error with increasing age. Subjects with cognitive decline had increased finger tapping variability and reduced ability to select the correct tapping fingers in the multi-finger tapping task compared to cognitively intact elderly subjects. Cognitively intact elderly subjects and those with cognitive decline had prolonged force release and reduced independence of finger movements compared to young adults and middle-aged adults. The findings suggest two different patterns of impaired manual dexterity: one related to cognitive decline and another related to healthy aging. Manual dexterity tasks requiring updating of performance, in accordance with (temporal or spatial) task rules maintained in short-term memory, are particularly affected in cognitive decline. Conversely, tasks requiring online matching of motor output to sensory cues were affected by age, not by cognitive status. Remarkably, no motor impairments were detected in patients with cognitive decline using clinical scales of hand function. The findings may have consequences for the development of manual dexterity markers of cognitive decline.

## Introduction

Cognitive aging represents the reduction of mental abilities with age, such as attention, memory function, and information processing speed (1). The prevalence of Mild Cognitive Impairment (MCI) and Alzheimer's disease (AD) increases strongly with age. These conditions are common in approximately 10% of the population over 65 years of age. And in about 50% of those over 85 years, who develop AD (2). Due to high prevalence of dementia in age, early detection and prediction of cognitive decline remains a key challenge in public health. Previous studies suggest a relationship between cognitive decline and impairments in hand motor function (3, 4). There is an increasing number of studies on sensorimotor markers of cognitive decline in AD and MCI. Sensorimotor performances have been investigated by several methods available in clinical settings like gait, postural equilibrium (5–7) or neuropsychological tests (8, 9). Markers of impaired manual dexterity have also been used (3), and a recent longitudinal 4-year cohort study found that prolonged time taken in two simple manual dexterity tasks (including putting on and buttoning a shirt) was related to higher risk of developing cognitive decline [according to MMSE; (10)]. Sensorimotor markers are considered independent of educational level (11), which is an advantage for clinical use. However, despite these promising results some issues remain unresolved. First, as a potential marker, what type of manual dexterity task and which type of performance variable is optimal? Performance measures previously used were most often global task-based measures, i.e., time taken to complete task (5, 6, 10). Thus, it remained unclear what aspect of sensorimotor control was being measured, making the rationale for detecting cognitive decline uncertain. A second issue, also relevant for comparing different sensorimotor markers, concerns the role of cognition in a given sensorimotor task. Most motor tasks also involve cognitive control such as attention, planning, prediction (12), and cognitive factors are increasingly being recognized as important for motor control (13, 14). Cognitive assessments, probing executive functions, can also be used to predict cognitive decline (8, 15). Improved detection of sensorimotor impairments in MCI patients has been found when assessed in a dual-task condition, with enhanced effect in counting tasks compared to verbal fluency tasks (5). Therefore, it is likely that sensorimotor performance measures incorporating cognitive control would enhance discrimination and improve detection of cognitive decline.

Manual dexterity is complex and can be defined as the ability to accurately and rapidly control finger movements in a coordinated and adaptive manner, such as fine control in grasping and manipulation of small objects. Manual dexterity is highly specialized in humans (16) allowing a rich repertoire of goal- and object-oriented manual control. Manual dexterity deteriorates with aging and can negatively impact activities of daily living and independence (17). Studies have reported age-related impairments in maximal grip force (18) sensory functioning (19, 20) and in grasping and manipulation of objects [Box and Block test (21, 22), NHPT (23, 24)]. Regarding specific manual dexterity components, accuracy in force control tasks is reduced in age (25, 26) and independence of finger movements may deteriorate (27). Increased variability of finger movements (28) and motor slowing (29) have also been documented. These studies suggest a complex multi-component decline in manual dexterity in older people, especially in the very old (30). However, it is less clear how age-related sensorimotor impairments relate to cognitive decline, and how those two compare. In particular, whether different measures of manual dexterity reflect sensorimotor or rather cognitive control has not been investigated so far.

The aim in this study was to use the Finger Force Manipulandum, developed for the measurement of multiple components of manual dexterity (31), to disentangle manual dexterity impairments due to cognitive decline from those related to age-related sensorimotor impairment (25). We hypothesized that manual dexterity tasks strongly dependent on executive functions (attention, working memory) would be differently affected by cognitive decline compared to tasks involving fewer cognitive constraints.

## Methods

### Participants

This cross-sectional observational study included four groups of participants recruited from Hôpital Pitié-Salpêtrière-Charles Foix, Paris and the Centre de Psychiatrie et Neurosciences, Paris. We studied three groups of healthy participants: young adults [YA, *N* = 10, 6F/4M, mean age ± *SD* = 26 ± 3 y, range (21–30 y)], middle-aged adults [MA, *N* = 8, 3F/5M, mean age = 41 ± 9y, (32–55 y)], cognitively intact elderly subjects [ES, *N* = 11, 7F/4M, mean age = 78 ± 8y, (68–93 y)] and one group of elderly subjects with cognitive decline [CD, *N* = 11, 8F/3M, mean age = 84 ± 7 y, (73–96 y)], consisting of either MCI or early Alzheimer's disease (AD). All participants reported being right-handed with a laterality quotient above than 0 according to the Edinburgh Handedness Inventory (32). Patients in the CD group had been previously diagnosed of MCI or early AD by an experienced geriatrician, accordingly to the National Institute on Aging—Alzheimer's Association criteria (33).

Exclusion criteria were any neurological, orthopedic, or age-related disorders that could affect their manual dexterity. A brief interview preceded all testing, to determine whether subjects met the inclusion criteria. Elderly subjects with cognitive decline also underwent additional clinical neuropsychological evaluation (see below).

Elderly subjects were participants of a larger study on health and functional recovery in a geriatric population post-transaortic valve implantation. Young and middle-aged adults were volunteers who underwent dexterity assessment for the purpose of another study. Ethical approval was obtained from local ethical committee (CPP, Ile de France). Informed consent was obtained from all participants and the study was conducted in accordance to the Declaration of Helsinki.

### Clinical measures

Upper extremity sensorimotor function was assessed in all elderly subjects (i.e., in healthy elderly and in subjects with cognitive decline) using the following tests. The Nine-Hole Peg Test [NHPT, (22)] was used to qualitatively evaluate precision grip and object manipulation. Both the dominant and non-dominant hands were tested twice, and the average time taken to place and remove all pegs of each hand was calculated. The Box and Blocks Test [BBT, (34)] was used to measure gross manual dexterity. The Jebsen Taylor hand function test [JTHFT, (24)] was used to evaluate fine and gross motor hand function. The pinch gauge [Patterson Medical Inc. (35)] was used to measure maximal strength in precision, key (lateral), palmar (three-jaw chuck) and pinch grips (best of three attempts recorded). Performance in right and left hands was measured in the participants. The Instrumental Activities of Daily Living Scale [IADL, (36)] as used to assess independent living skills. Patients were scored according to their highest level of functioning using a summary score that ranges from 0 (low function, dependent) to 14 (high function, independent) (37). Sensory function was tested through light touch-test (Semmes-Weinstein Monofilaments). This provided an evaluation of cutaneous sensitivity of finger tips (38).

Neuropsychological assessments were performed by a neuropsychologist. It included the Mini-Mental State Examination (MMSE) and a more detailed neuropsychological assessment to document the presence or absence of cognitive decline. Neuropsychological testing included in most cases the Dubois test of verbal episodic memory (39), the French version of the Free and cued selective reminding test [RL/RI-16; (40)], the French version of the Listening Span Test (EMPANS), the French version of the Frontal Assessment Battery [Batterie rapide d'efficience mentale, BREF; (41)], the Verbal Fluency Test, which assesses semantic memory (42), as well as the figure of Rey test.

### Finger force manipulandum tasks

**Figure 1:**
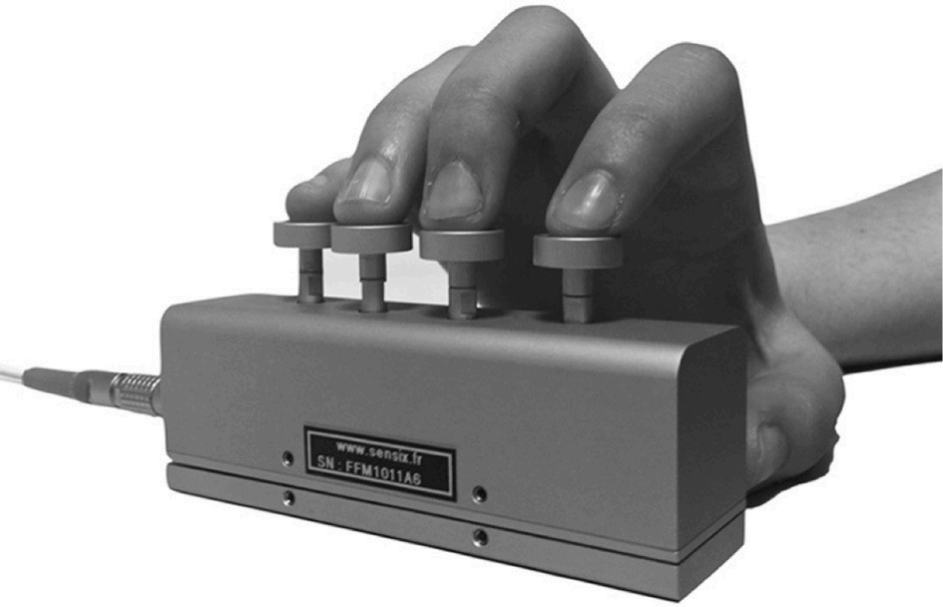
The Finger Force Manipulandum (FFM). Index, middle, ring, and little finger each apply forces on separate spring-loaded pistons. In the force tracking task, graduated force was exerted on one piston (index finger). In single and multi-finger tapping tasks the subject was instructed to tap on the corresponding piston(s) in response to auditory or visual cues without trying to match a particular force level (no force constraint).

**Figure 2:**
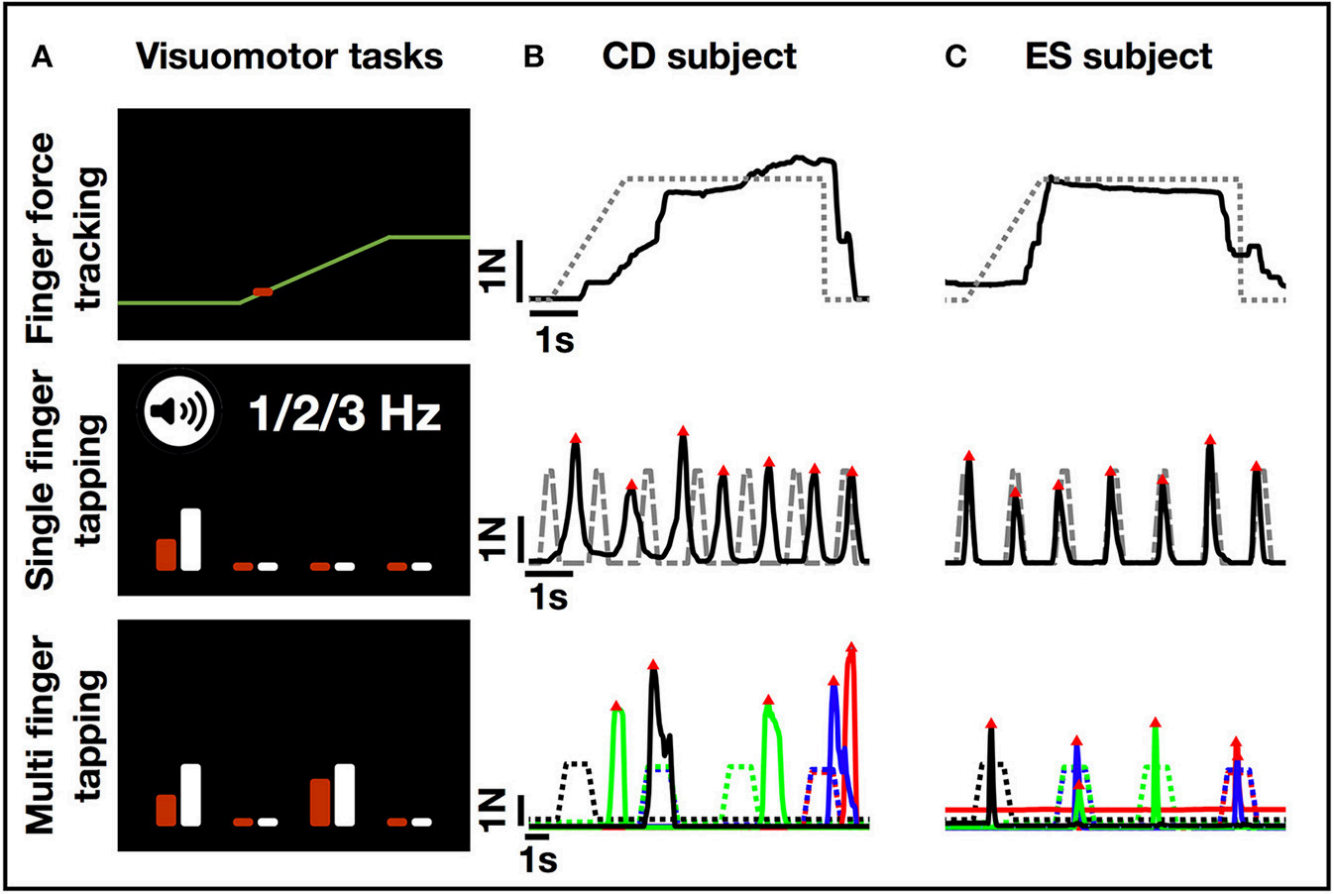
FFM task display and single trial performance examples. **(A)** Examples from the visual display of the three FFM tasks. In the finger force tracking task, the subject matched the force applied on the piston (represented as a red cursor that moves vertically as a function of force) to the target force trajectory (yellow right-to-left moving line) displayed on computer screen. In the single finger tapping task, the subject performed repeated tapping with a single finger following auditory cues at a given rate. The white bar on the screen indicated which finger had to perform the tapping task (here the index finger), while the red bar gave a visual feedback of which finger is being selected by the subject. The length (height) of each bar was a function of force. In the multi-finger tapping task, the subject was instructed to perform a one or two-finger tap with fingers matching the visual cues on the screen (here the instruction indicates a two-finger tap using the index and ring finger). **(B)** Single trial recordings from a subject in the cognitive decline (CD) group. **(C)** Single trial recordings from a subject in the elderly subjects (ES) group. Note: greater variability in single finger tapping and difficulty selecting correct finger to tap with in multi-finger tapping task (the performed taps do not match the cues indicated by the stippled line). Color code: blue, index; red, middle; green, ring; black, little finger. The four trials from left to right represent: a single (little) finger tap, followed by a two-finger (index, little) tap, a single (ring) finger tap, and another two-finger (index, middle) tap.

Manual dexterity components were measured using the Finger Force Manipulandum (FFM; http://www.sensix.fr^1^), a device with force sensitive pistons linked to various visuo-motor tasks [Figure 1, (31)]. Individual force data for each finger (index, middle, ring and little finger) were sampled at 10 KHz using a CED1401® (www.ced.co.uk) connected to a computer running Spike2V6®, which provided real-time visual display of finger forces together with target instructions. Three FFM tasks, as described previously (43) were used (Figure 2A):

- The finger force tracking task was used to measure precision of index fingertip force modulation. Subjects were instructed to accurately match the applied index finger force to the target force. The applied force was displayed in real-time as a cursor moving vertically as a function of force. The target force was displayed by a moving line. Each trial was composed of a ramp (linearly increasing force), a hold (static maintenance of force), and a release phase (instantaneous drop in target force back to baseline level, 0N). Trials were separated by 3 s rest. A total of 48 trials were performed in eight blocks (four with 1N and four with 2N target hold force) in alternating order.
- The single finger tapping task was used to measure performance of rhythmic tapping at 1, 2, and 3 Hz. For each finger, subjects were instructed first to follow auditory rhythmic cues by tapping on the piston. After 15 auditory-cued trials, subjects had to continue tapping 15 trials at the same rate without auditory cues (total number of trials per frequency for each finger: 30).
- The multi-finger tapping task was used to measure the independence of finger movements. Subjects were instructed to reproduce different finger tap combinations according to displayed target instructions within a 2 s time window. Trials consisted of single finger taps (separate tap of index, middle, ring or little finger; each performed 8 times for a total of 32 single finger trials) or two-finger tap combinations (simultaneous taps of index-middle, index-ring, index-little, middle-ring, middle-little, or ring-little fingers; each performed 5 times for a total of 30 two-finger trials). The sequence of trials was pseudo-randomized.

### Data analysis

Visuomotor performance was analyzed using MatlabV9.1 (The MathWorks, Inc., Natick, MA, USA). Raw data of the four finger force signals was first down-sampled to 100 Hz (and then smoothed using a 20 ms sliding window). The following measures were first extracted trial-by-trial and then averaged across trials for each task and condition (e.g., for a single subject in CD and ES groups in Figures 2B,C).

- Finger force tracking:
  - Tracking error (N) was calculated as the absolute summed error between the ideal target force and the user applied force. The tracking error was extracted separately in the ramp and hold phase for each trial (total of 48 trials).
  - Release duration (ms) was calculated as the time taken to instantaneously reduce the user-applied force from 75 to 25% of the target force (total of 48 trials).

The data of the two tapping tasks were analyzed with a peak detection algorithm allowing identification of finger taps of a minimal force amplitude (>0.5N). All detected taps were then categorized as correct (detected tap = target instruction) or incorrect (detected tap ≠ target instruction). Incorrect taps included “overflow taps” (presence of unwanted extra finger tap while correctly matching the target finger) and “error taps” (presence of unwanted extra finger tap in absence of a correct finger tap). The following task-specific measures were calculated:

- Single finger tapping:
  - Tap frequency: mean tapping frequency (Hz) performed during 1, 2, or 3 Hz conditions during auditory cues (15 taps) or without auditory cues (equivalent time).
  - Standard deviation (SD) Tap interval: tap interval (ms) variability between two successive finger taps during 1, 2, or 3 Hz condition.
- Multi-finger tapping:
  - Selectivity index: rate (%) of correct finger taps matching the target (non-target taps were not considered).
  - Individuation index: rate (%) of correct finger taps matching the target in absence of incorrect taps.

### Statistical analysis

Statistical analyses of clinical and behavioral measures were performed using Statistica10 (StatSoft, Inc., USA). Student's *t*-test or Mann-Whitney *U*-test were used to test for group differences in demographic and clinical outcomes. Group differences of FFM measures were analyzed using a general linear model repeated measures ANOVA with one GROUP factor (YA/A/ES/CD) and task-related within-group factors:

- Finger force tracking: FORCE (1N and 2N) and PHASE (RAMP and HOLD)
- Single finger tapping: FREQUENCY (1, 2, and 3 Hz), FINGER (index, middle, ring, little finger), PHASE (auditory-cued, without feedback)
- Multi-finger tapping: FINGER (index, middle, ring, little finger), COMBINATION (single and two-finger taps)

Fisher LSD *post-hoc* test was used to investigate differences revealed by ANOVA.

## Results

Demographic and clinical details of elderly subjects and patients with cognitive decline are shown in Table 1.

**Table 1:** Clinical data for the two groups: elderly subjects (ES) and patients with cognitive decline (CD).

	**MEAN** ±**SD**		
	**Cognitively intact elderly subjects (ES)**	**Elderly subjects with cognitive decline (CD)**	Group difference^*^
Age (years)	78.20 ± 8.47	83.64 ± 6.85	*p* = 0.12
MMSE (0–30)	27.25 ± 3.20	22.36 ± 3.59	***p*** = **0.007**
IADL (0–14)	10.33 ± 2.90	11.18 ± 3.52	*p* = 0.533
BBT right (#blocks/ min)	47.67 ± 14.09	43.36 ± 10.59	*p* = 0.45
BBT left (#blocks/ min)	41.92 ± 14.5	42.91 ± 10.06	*p* = 0.852
JTHFT right (s)	47.5 ± 13	49.1 ± 9.6	*p* = 0.75
JTHFT left (s)	55.5 ± 17.3	55.6 ± 13.6	*p* = 0.99
Pinch right (Kg)	8.23 ± 2.98	7.8 ± 2.26	*p* = 0.70
Pinch left (Kg)	7.27 ± 2.37	6.77 ± 1.96	*p* = 0.60
NHPT right (s)	29.35 ± 10.74	27.67 ± 2.41	*p* = 0.675
NHPT left (s)	33.05 ± 10.45	33.21 ± 6.24	*p* = 0.995

*MMSE, Mini-Mental State Examination (min = 0, max = 30, score ≥ 24 normal, score 18-23 MCI) (44); IADL, Instrumental Activities of Daily Living Scale (45); BBT, Box and Blocks Test (34) performed with right and left hand, respectively; JTHFT, Jebsen-Taylor Hand Function Test (24) total score across six tasks; Pinch Gauge instructions for tip, key and palmer pinch strength, average reported (35); NHPT, Nine-Hole Peg Test (22) finger dexterity test*.

* *Group difference tested using Mann-Whitney U-test. Significant differences are highlighted in bold*.

### FFM task feasibility

All subjects successfully performed the finger force tracking task. Two participants in the elderly subjects group were not able to complete the single and multi-finger tapping tasks due to limited time or unwillingness to complete the full protocol. In the cognitive decline group, four participants were not able to complete the multi-finger tapping task due to an inability to use the visual feedback in the allotted time window [task-related issue similar to (31)].

### Group comparisons for dexterity components

#### Finger force tracking

**Figure 3:**
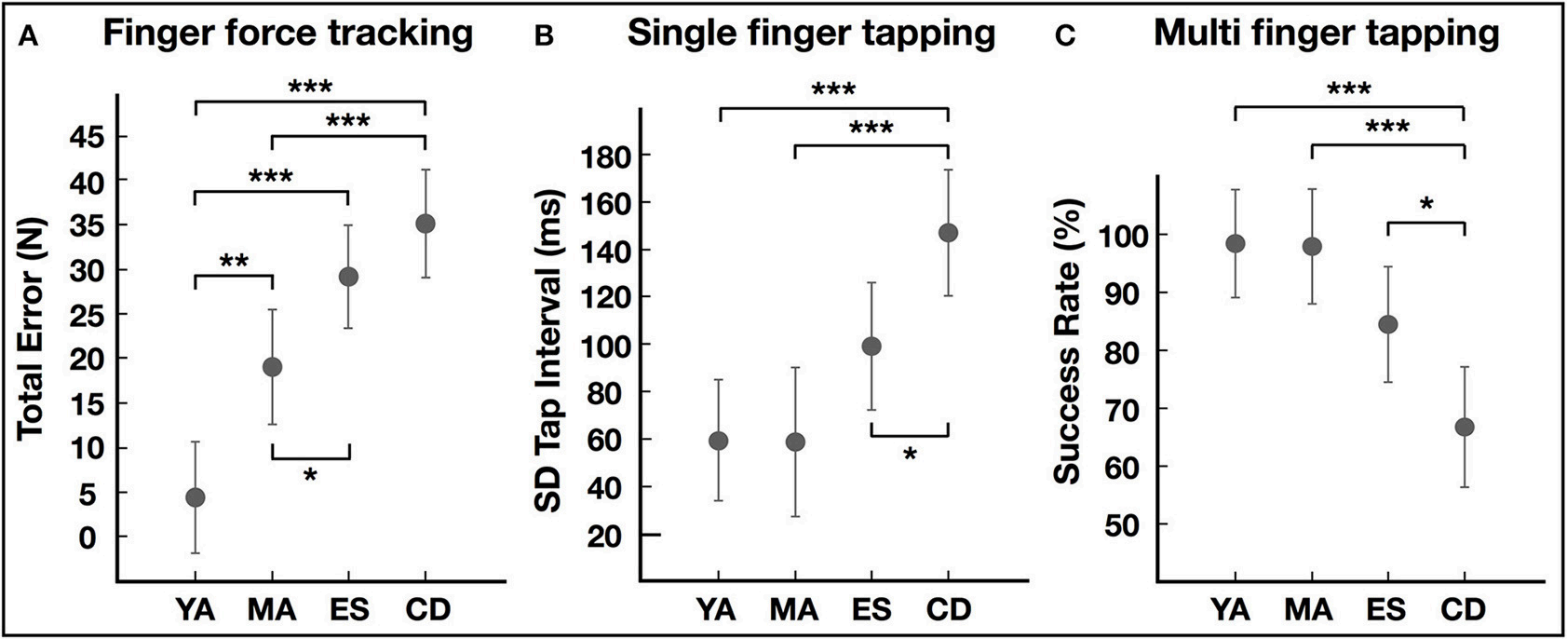
Group comparisons of performance in FFM tasks. **(A)** Error during finger force tracking task. **(B)** Variability of intertap interval in single finger tapping task. **(C)** Success rate (also termed the selectivity index) in the multi-finger tapping task. YA, young adults; MA, middle-aged adults; ES, elderly subjects; CD, subjects with cognitive decline. Group differences in LSD *post-hoc* tests: *p < 0.05, **p < 0.01, ***p < 0.001.

Qualitatively, this task revealed striking differences in the ability to precisely control forces with increasing age. Single subject/single trial examples are shown in Figure 2A. The ANOVA of tracking error showed significant group differences [*F*_(3, 36)_ = 18.60, *p* < 0.001, Figure 3A]. *Post-hoc* testing revealed that young adults (YA) had smaller errors compared to other groups (Table 2, *p* < 0.05). Young adults also had decreased error compared to elderly subjects (*p* = 0.03) and to subjects with cognitive decline (*p* = 0.001). However, error did not differ between elderly subjects and those with cognitive decline (*p* = 0.16). Release duration also changed as a function of age [GROUP *F*_(3, 36)_ = 3.18, *p* = 0.02). *Post-hoc* testing showed that both elderly subjects and those with cognitive decline had increased release duration compared to young adults (Table 2, *p* < 0.05).

**Table 2:** FFM measures for the four groups (mean ± standard deviation).

		**MEAN** ±**SD**				**Group differences**		
		**Young adults (YA)**	**Middle-aged adults (MA)**	**Elderly subjects (ES)**	**Subjects with cognitive decline (CD)**	**YA vs. MA**	**MA vs. ES**	**ES vs. CD**
Finger force tracking	Tracking error (N)	4.44 ± 0.91	19.01 ± 3.68	29.54 ± 10.84	35.82 ± 15.81	***p*** = **0.005**	***p*** = **0.03**	*p* = 0.16
	Release duration (ms)	84.03 ± 33.60	84.03 ± 33.60	237.58 ± 151.74	198.16 ± 147.33	*p* = 0.56	***p*** = **0.03**	*p* = 0.43
Single finger tapping (3 Hz)	Frequency (Hz)	3.26 ± 0.30	3.12 ± 0.46	2.49 ± 0.46	2.49 ± 0.46	*p* = 0.31	***p*** <**0.001**	*p* = 0.19
	SD tap interval (ms)	59.54 ± 20.30	59.11 ± 16.01	98.91 ± 38.94	146.25 ± 72.09	*p* = 0.98	*p* = 0.08	***p*** = **0.03**
Multi-finger tapping	Selectivity Index (%)	99.17 ± 0.93	98.88 ± 1.52	84.53 ± 11.25	66.11 ± 33.93	*p* = 0.96	*p* = 0.07	***p*** = **0.03**
	Individuation index (%)	93.82 ± 4.57	93.19 ± 3.68	61.17 ± 28.78	50.05 ± 37.69	*p* = 0.95	***p*** = **0.002**	*p* = 0.28

*LSD Fisher post-hoc tests were used to evaluate significant group effects in ANOVA. Significant differences are highlighted in bold*.

#### Single finger tapping

The ANOVA of tap frequency showed GROUP differences [*F*_(3, 34)_ = 9.94, *p* < 0.001] and significant interaction with frequency conditions [GROUP^*^FREQ, *F*_(6, 68)_ = 11.07, *p* < 0.001]. *Post-hoc* testing revealed that all groups performed similarly at 1 Hz (YA: 1.06 Hz ± 0.04; MA: 1.02 Hz ± 0.03; ES: 1.09 Hz ± 0.14; CD: 1.02 Hz ± 0.14) and 2 Hz (YA: 2.07 Hz ± 0.12; MA: 2.01 Hz ± 0.08; ES: 1.80 Hz ± 0.28; CD: 1.74 Hz ± 0.19). However, at 3 Hz elderly subjects as well as subjects with cognitive decline had reduced tap frequency compared to young adults and middle-aged adults (*p* < 0.001), detailed in Table 2. No difference was found between elderly subjects and those with cognitive decline.

The variability of tapping at 3 Hz also differed significantly between groups [ANOVA, GROUP, *F*_(3, 34)_ = 8.44, *p* < 0.001, Figure 3B]. *Post-hoc* tests showed no effect of age and only the subjects with cognitive decline had significantly increased tap interval variability compared to the other groups (Table 2, *p* < 0.05).

#### Multi finger tapping

The ANOVA of the selectivity index showed significant GROUP differences [*F*_(3, 28)_ = 6.91, *p* = 0.001, Figure 3C]. Only the cognitive decline group had greater difficulty to tap with the correctly selected finger compared to the other groups (Table 2, *p* < 0.05). Furthermore, the ANOVA showed a significant interaction between GROUP and COMBINATION [*F*_(3, 28)_ = 4.90, *p* = 0.007]. *Post-hoc* testing revealed that the performance of elderly subjects did not differ from those of young adults (*p* = 0.16) or middle-aged adults (0.19) when tapping with one finger. The selectivity of taps was reduced in elderly subjects when tapping with two fingers (ES^*^YA, p = 0.02; ES^*^MA, *p* = 0.02). In contrast, the cognitive decline group had reduced selectivity compared to all groups in both one and two-finger taps.

The individuation index also varied between groups [GROUP *F*_(3, 28)_ = 10.30, *p* < 0.001] but there was no interaction between GROUP × COMBINATION [*F*_(3, 28)_ = 1.50, *p* = 0.24]. Thus, elderly subjects and those with cognitive impairment showed a significantly decreased group performance compared to young adults and middle-aged adults (Table 2, *p* < 0.05). Furthermore, this index showed no significant differences between elderly subjects and those with cognitive impairment.

### Clinical measures of hand sensory and motor impairment and ADL

Clinical measures were obtained for all elderly subjects, i.e., for healthy elderly subjects and those with cognitive impairment. Sensory function (light touch) was normal in all subjects. The MMSE score was, as expected, significantly lower in the subjects with cognitive impairment compared to elderly subjects. However, the mean MMSE score of 22.36 in the cognitive impairment group indicated the absence of a major cognitive disorder. For the clinical manual dexterity tests (pinch grip strength, gross and fine manual dexterity; see Table 1) elderly subjects and those with cognitive impairment showed comparable performance (no significant group differences). In both of these groups no dependency in activity of daily living was observed.

## Discussion

This study provides a first multi-component characterization of changes in manual dexterity related to aging and to cognitive decline. The behavioral data suggest two different patterns of deterioration in manual dexterity. (i) The first pattern of impaired performance was found in patients with a medical history of cognitive decline. These patients had increased variability of finger tapping and reduced ability to correctly select a finger in response to a visual target in the multi-finger tapping task. Remarkably, impaired performance was only related to cognitive status and not to increasing age. The second pattern of decline in performance was related to increasing age and was present in tasks that required fine-graded sensorimotor processing, such as visuo-motor precision during force tracking, finger tapping rate at 3 Hz and individuation of finger movements. Independent finger movements are considered a hallmark of manual dexterity (46), and we found impaired independence of finger movements in elderly subjects. These pilot findings need to be confirmed in larger samples. Nonetheless, the two patterns of impaired performance suggest a dissociation between manual tasks involving mainly sensorimotor processing (sensorimotor integration, speed of execution and motor inhibition) and those involving a greater cognitive contribution (attention and working memory).

### Cognitive effects

Two specific impairments were only detected in subjects with cognitive decline. First, tap interval variability, in the *audio-motor* single finger tapping task, was higher (Figure 3B), replicating previous findings of increased intra-individual variability in finger tapping in MCI (47–49). Increased tapping variability in MCI is considered to arise from impaired working memory and attentional processing (48, 49), not from impaired motor control. Furthermore, increased tapping variability has also been shown in patients with attention deficit hyperactivity disorder (50). Impaired working memory and attention may compromise matching of internal task expectancies with external temporal demands (51) or affect task planning (52) and prediction (53). Second, subjects with cognitive decline showed reduced ability to select the correct finger according to the visual cue during the multi-finger tapping task (Figure 3C). This was not the case in healthy middle-aged and young adults, suggesting absence of an age effect. This task resembles the serial reaction time task (SRTT), which revealed prolonged processing times in MCI (9, 54, 55). Although older healthy subjects showed prolonged reaction times in SRTT, they had similar error rates compared to young subjects (56), coherent with our findings of similar selectivity index in healthy subjects of different age. Selecting the correct finger (effector), in response to the visual cue, requires spatial mapping between cue and effector according to rules maintained in short-term memory (57). This stimulus-response relation and selection process is likely affected in MCI, consistent with impaired associative memory and decision making (58–60).

### Aging effects

A strong age effect was found in the ability to precisely match finger force to a visual target in the force tracking task. Error increased linearly with age across groups, with elderly subjects and subjects with cognitive decline having the highest error values (Figure 3A). Even the group of adults had increased error compared to the group of young adults, providing evidence of an early age-related decline in the precision of sensorimotor control. This shows that the capacity to adapt motor performance in accordance to visual feedback deteriorates with age. Previous studies showed a two-fold increase in force-tracking error in subjects 60–70 years old compared to young subjects (age ~20) (61, 62). Our results extend these findings, showing an even greater decline (~6-fold) in force tracking precision in elderly subjects and subjects with cognitive decline (age >70). Importantly, subjects with cognitive decline did not perform worse than elderly subjects of comparable age, suggesting that MCI does not impact visuomotor force tracking, which relies primarily on on-line sensorimotor integration, less on cognitive resources. This is consistent with absence of visuo-motor upper limb deficits in MCI, as long as cognitive demands (mapping, memory, learning) are minor (63).

Our findings also suggest an age-related decline in motor inhibition: longer release duration (during tracking) and reduced finger individuation, both considered to involve processes of motor inhibition (31, 64, 65), were found in elderly subjects and those with cognitive decline. Age-related increase in release duration has been reported previously in healthy subjects (62), whereas altered finger individuation has not been shown consistently (27, 66), probably due to task-related differences. Our tapping task, resembling keyboard typing or piano playing, revealed evidence for a similar reduction of independent finger movements in elderly subjects and, in subjects with cognitive decline. This suggests that MCI does not influence these measures linked to motor inhibition, in line with previous reports using Go-Nogo paradigms or Stroop (67).

Our data also point to age-related motor slowing. Both elderly subjects [as those in (68)] and those with cognitive decline were unable to maintain single finger tapping at 3 Hz, but showed no speed deficit at slower tapping rates (1 or 2 Hz). Tapping speed did not differentiate between elderly subjects and those with cognitive decline, similar to previous reports (69–71).

### Differential cognitive involvement in manual dexterity tasks

**Figure 4:**
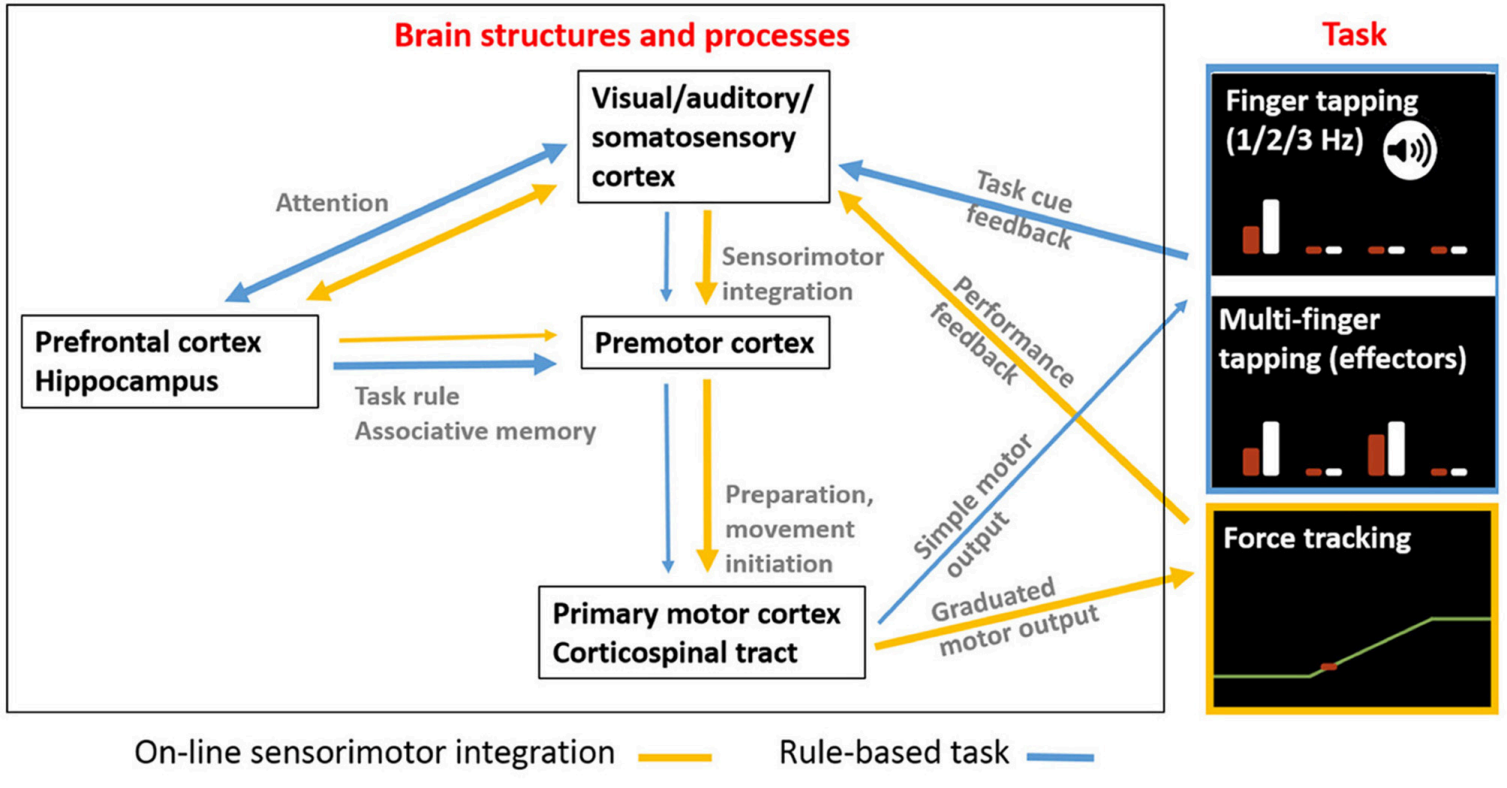
A hypothetical overview of how different brain areas are more or less involved depending on manual dexterity tasks involving more cognitive rule-making or more sensorimotor integration requirements. Arrow thickness reflects degree of task-related involvement processes. The hypothesis is that brain areas and processing involved in manual dexterity tasks depend on whether the task requires (i) on-line sensorimotor integration (yellow arrows) to adapt performance according to feedback or (ii) rule-based associations (blue arrows) that result in performance predictions that are adapted during performance. The force tracking task requires a greater level of on-line matching of motor output to sensory feedback with engagement of sensory-premotor-motor networks. In contrast, tasks requiring more cognitive processing including stimulus-based decisions (temporal or spatial) according to task rules (maintained in working memory) involve a greater contribution from prefrontal cortex and hippocampus. The basal ganglia and the cerebellum, also involved in manual dexterity processing, are not shown for simplicity.

We propose a qualitative, explanatory model that accounts for the observed differences in manual dexterity as a function of *cognitive decline* vs. *healthy aging*. The model incorporates (i) the cognitive and sensorimotor constraints of each task, and (ii) the presumably involved brain structures and processes (Figure 4).

- *Task constraints*: the single and multi-finger tapping tasks both require a mapping between motor performance and stimulus-based rules. In the single-finger tapping task the rule involves tapping (with one finger at a time) in synchrony with the auditory cue and then continuing without cue. Single finger tapping is thus the one task among the three that contains an explicit memory condition (other than task instructions) on the timing of repetitive motor action, which determines the degree of performance. In the multi-finger tapping task the rule requires mapping the visually displayed target tap to the effector configuration. Thus, finger selection based on this trial-by-trial mapping determines here successful task performance. In these two tasks, specific rule-based (temporal or spatial) information is maintained in short-term memory. Attention and working memory processes are therefore key to good performance in these two tasks, that do not require high-level sensorimotor integration. In contrast, the force-tracking task requires constant on-line modulation of finger force based on real-time visual feedback (sensorimotor integration), but depends, most likely, less on working memory (since the feedback is available at all times and the task rules are simple and invariant).
- *Neural correlates*: we presume that implementation of (temporal or spatial) stimulus-response rules, requiring attention and working memory, depend on prefrontal cortical areas and hippocampus (72). This concerns primarily the two tapping tasks. In contrast, we assume that force tracking, requiring a high degree of visuo-motor integration, depends predominantly on sensorimotor (parieto-motor) cortical networks (73, 74).

The proposed model is compatible with studies suggesting a disassociation of neural mechanisms related to age-related sensorimotor deterioration or cognitive decline. Neuroimaging studies provide compelling evidence of reduced structural and functional integrity of prefrontal cortex and hippocampus in patients with MCI (72, 75), which has been correlated with gait slowing and cognitive dysfunction in elderly subjects (76). Decision making and response selection are closely linked to the prefrontal cortex (77–79), which is dysregulated in MCI subjects (80). In contrast, age-related decline in motor function has been mainly related to loss of structural and functional integrity in descending motor and ascending sensory pathways (20, 25, 81, 82). Furthermore, healthy aging has been related with increased recruitment of prefrontal and sensorimotor networks to successfully accomplish more cognitively demanding motor tasks (83). Our results suggest that elderly subjects affected by MCI cannot use compensatory cognitive reserves, consistent with decreased performance in more cognitively demanding tasks (81, 84, 85).

### Limitations

The cognitive decline group consisted of a heterogeneous sample of patients with clinical MCI or early stage AD diagnosis. Group size was small and sub-group characterization was not feasible. Nonetheless, this study provides a first multi-component description of dexterity in patients with cognitive decline. Future studies with a larger sample would be needed to (i) assess the presence of different manual dexterity profiles in various types of MCI and AD, (ii) replicate the absence of an age effect in particular key dexterity scores, and (iii) include a finger motor sequence (memorization) task (43) to potentially evaluate differences between amnestic and non-amnestic types of MCI (86).

## Conclusions

Although conventional clinical testing of hand function (BBT, 9-HPT, JTHFT) did not reveal any differences between elderly subjects with cognitive decline and those without, the quantitative assessment of manual dexterity showed clearly distinct task performance. Subjects with cognitive impairment showed decreased single-finger tapping regularity and reduced finger selectivity (compared to healthy elderly, age-matched subjects). In contrast, accuracy of force control was significantly reduced with age (even between young adults and adults), but not more so in subjects with cognitive decline. This dissociation suggests that rule-based dexterity tasks are useful for the detection of MCI and that on-line sensorimotor integration tasks are sensitive for determining age-related decline in manual dexterity in healthy subjects. Furthermore, our findings imply that these performance measures, suitable for rapid quantification in the clinical setting, could provide valuable clinical markers for early sensitive detection of age-related cognitive decline. Further studies in longitudinal cohorts are warranted to investigate whether these measures could be useful for predicting the development of MCI (87).

## Author contributions

PL, JB, and MM conceptualization, LC, AA, and PL formal analysis, LC and AA investigation, PL, JB, MM, and CL-L methodology, SP and JB resources, PL and JB supervision, LC, AA, and PL writing—original draft, LC, AA, PL, JB, MM, CL-L, and SP writing—review and editing.

### Conflict of interest statement

PL owns shares in Aggero MedTech AB, a company commercializing a measurement instrument for spasticity. PL and MM have patented a method for measurement of manual dexterity (EP2659835A1), but do not own commercialization rights. JB reports fees or invitations unrelated to this work from Boehringer Ingelheim, GlaxoSmithKline, MSD, Amgen, Novartis, Sanofi Aventis, Pfizer, and Santor Edition. The remaining authors declare that the research was conducted in the absence of any commercial or financial relationships that could be construed as a potential conflict of interest.

## Footnotes

^1^SENSIX FORCE-TORQUE SENSOR FOR BIOMECHANICS Available online at: http://www.sensix.fr/ (Accessed June 3, 2018).
